# Supplementary material for: Early innate immune signatures correlate with Ad26.COV2.S vaccine durability and protective efficacy
Source: bioRxiv. 2026 Jun 10:2026.06.09.731069. Preprint. [Version 1] doi: 10.64898/2026.06.09.731069 (PMC13278152; doi:10.64898/2026.06.09.731069)
Supplement: Supplementary file 1 [file NIHPP2026.06.09.731069v1-supplement-1.pdf]

bioRxiv preprint doi: https://doi.org/10.64898/2026.06.09.731069; this version posted June 10, 2026. The copyright holder for this preprint (which was not certified by peer review) is the author/funder, who has granted bioRxiv a license to display the preprint in perpetuity. It is made available under a CC-BY 4.0 International license.

Figure S1: Whole-blood innate immune transcriptomic signatures at day 1 following Ad26.COV2.S boost in participants demonstrate moderate yet largely unsignificant predictive ability of antibody and T cell responses 238 days following initial immunization. A Spearman-rank test was used to calculate correlations, and *p*-values were adjusted for multiple comparisons using a Bonferroni correction. From left to right, signatures are

bioRxiv preprint doi: https://doi.org/10.64898/2026.06.09.731069; this version posted June 10, 2026. The copyright holder for this preprint (which was not certified by peer review) is the author/funder, who has granted bioRxiv a license to display the preprint in perpetuity. It is made available under a CC-BY 4.0 International license.

correlated to: CD4+ T cell activity measured by intracellular staining (**ICS**), CD8+ T cell activity measured by **ICS**, anti-RBD antibody responses (**ELISA**), T cell activity (**ELISPOT**), and **NT50** titers.

Figure S2: Whole-blood innate immune transcriptomic signatures at day 7 following Ad26.COV2.S boost in participants demonstrate no predictive ability of antibody and T cell responses 238 days following initial immunization. A Spearman-rank test was used to calculate correlations, and *p*-values were adjusted for multiple comparisons using a Bonferroni correction. From left to right, signatures are correlated to: CD4+ T cell activity measured by intracellular staining (**ICS**), CD8+ T cell activity measured by **ICS**, anti-RBD antibody responses (**ELISA**), T cell activity (**ELISPOT**), and **NT50** titers.

Supplementary Figure 1

Transcriptomic Signatures Correlations - Human D1 Post-Boost Pathways vs. D239 Post-Prime Immunological Outcomes for Washington, Delta, and Omicron Variants

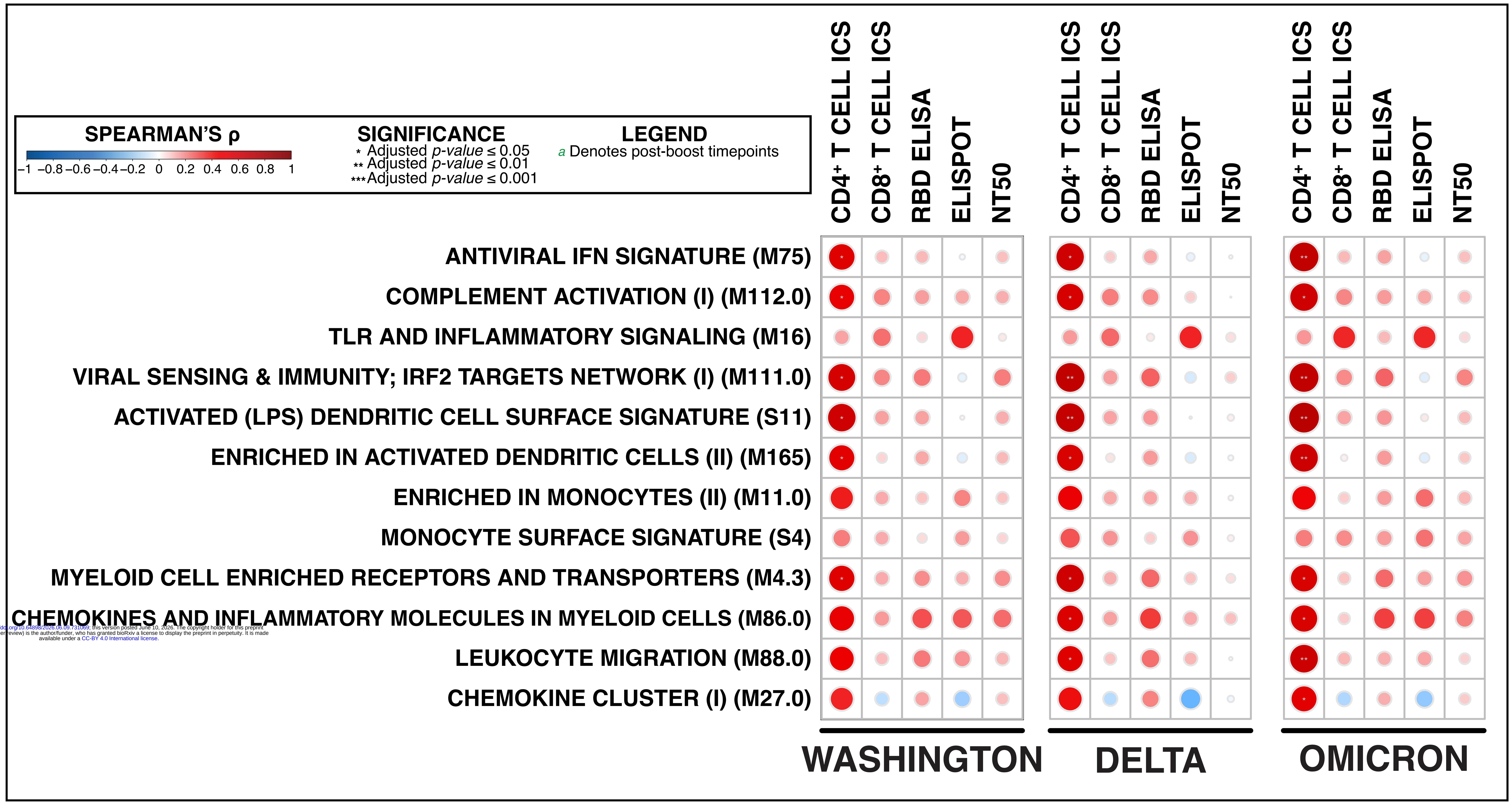

bioRxiv preprint doi: https://doi.org/10.64898/2026.06.09.731069; this version posted June 10, 2026. The copyright holder for this preprint (which was not certified by peer review) is the author/funder, who has granted bioRxiv a license to display the preprint in perpetuity. It is made available under a CC-BY 4.0 International license.

Supplementary Figure 2

Transcriptomic Signatures Correlations - Human D7 Post-Boost Pathways vs. D239 Post-Prime Immunological Outcomes for Washington, Delta, and Omicron Variants

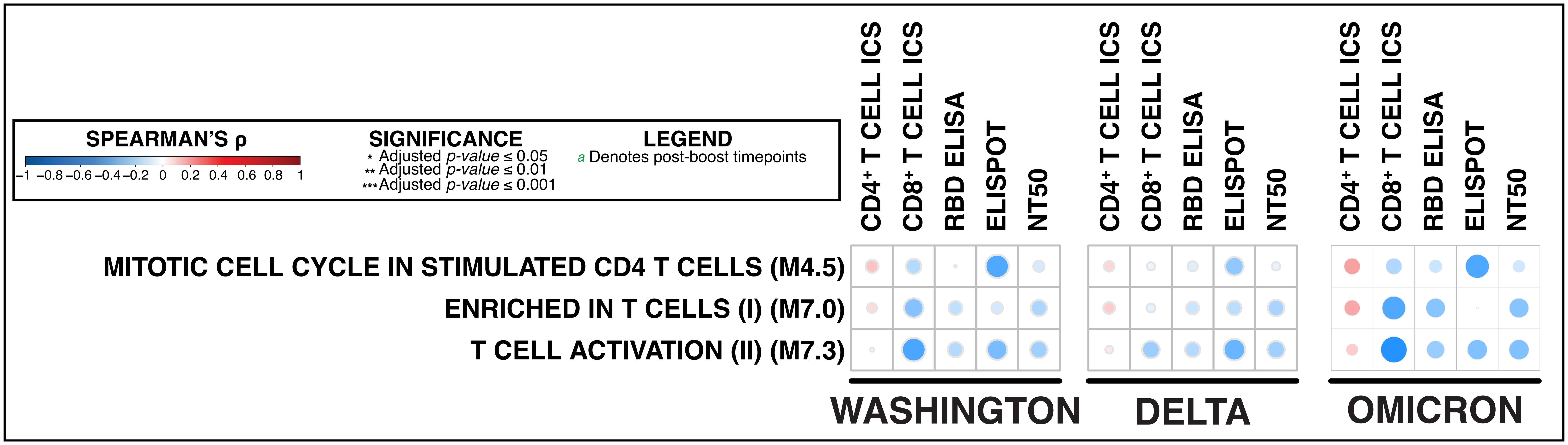

bioRxiv preprint doi: https://doi.org/10.64898/2026.06.09.731069; this version posted June 10, 2026. The copyright holder for this preprint (which was not certified by peer review) is the author/funder, who has granted bioRxiv a license to display the preprint in perpetuity. It is made available under a CC-BY 4.0 International license.
